# Genomic characterization of Huntington’s disease genetic modifiers informs drug target tractability

**DOI:** 10.1093/braincomms/fcae418

**Published:** 2025-01-11

**Authors:** Kevin Lucy Namuli, Alana N Slike, Mason A Hollebeke, Galen E B Wright

**Affiliations:** Department of Pharmacology and Therapeutics, Max Rady College of Medicine, Rady Faculty of Health Sciences, University of Manitoba, Winnipeg, MB, CanadaR3E 0T6; PrairieNeuro Research Centre, Kleysen Institute for Advanced Medicine, Health Sciences Centre and Rady Faculty of Health Sciences, University of Manitoba, Winnipeg, MB, CanadaR3E 3J7; Department of Pharmacology and Therapeutics, Max Rady College of Medicine, Rady Faculty of Health Sciences, University of Manitoba, Winnipeg, MB, CanadaR3E 0T6; PrairieNeuro Research Centre, Kleysen Institute for Advanced Medicine, Health Sciences Centre and Rady Faculty of Health Sciences, University of Manitoba, Winnipeg, MB, CanadaR3E 3J7; Department of Pharmacology and Therapeutics, Max Rady College of Medicine, Rady Faculty of Health Sciences, University of Manitoba, Winnipeg, MB, CanadaR3E 0T6; PrairieNeuro Research Centre, Kleysen Institute for Advanced Medicine, Health Sciences Centre and Rady Faculty of Health Sciences, University of Manitoba, Winnipeg, MB, CanadaR3E 3J7; Department of Pharmacology and Therapeutics, Max Rady College of Medicine, Rady Faculty of Health Sciences, University of Manitoba, Winnipeg, MB, CanadaR3E 0T6; PrairieNeuro Research Centre, Kleysen Institute for Advanced Medicine, Health Sciences Centre and Rady Faculty of Health Sciences, University of Manitoba, Winnipeg, MB, CanadaR3E 3J7

**Keywords:** monogenic disease, human genetics, repeat expansion

## Abstract

Huntington’s disease is caused by a CAG repeat in the *HTT* gene. Repeat length correlates inversely with the age of onset but only explains part of the observed clinical variability. Genome-wide association studies highlight DNA repair genes in modifying disease onset, but further research is required to identify causal genes and evaluate their tractability as drug targets. To address these gaps and learn important preclinical information, we analysed genome-wide association study data from a large Huntington’s disease age-of-onset study (*n* = 9064), prioritizing robust candidate Huntington’s disease modifier genes using bioinformatic approaches and analysing related information for these genes from large-scale human genetic repositories. We supplemented this information with other Huntington’s disease–related screens, including exome studies of Huntington’s disease onset and high-throughput assessments of mHTT toxicity. To confirm whether Huntington’s disease modifiers are shared across repeat expansion disorders, we also analysed age-of-onset genome-wide association study data from X-linked dystonia-parkinsonism caused by a (CCCTCT)_n_ expansion. We also studied modifier-related associations with rare diseases to inform potential off-target therapeutic effects and conducted comprehensive phenome-wide studies to identify other traits linked to these genes. Finally, we evaluated the aggregated human genetic evidence and theoretical druggability of the prioritized Huntington’s disease modifier genes, including characteristics recently associated with clinical trial stoppage due to safety concerns (i.e. human genetic constraint, number of interacting partners and RNA tissue expression specificity). In total, we annotated and assessed nine robust candidate Huntington’s disease modifier genes. Notably, we detected a high correlation (*R*^2^ = 0.78) in top age-of-onset genome-wide association study hits across repeat expansion disorders, emphasizing cross-disorder relevance. Clinical genetic repositories analysis showed DNA repair genes, such as *MLH1*, *PMS2* and *MSH3*, are associated with cancer phenotypes, suggesting potential limitations as drug targets. *LIG1* and *RRM2B* were both associated with neurofibrillary tangles, which may provide a link to a potential role in mHTT aggregates, while *MSH3* was associated with several cortical morphology-related traits relevant to Huntington’s disease. Finally, human genetic evidence and theoretical druggability analyses prioritized and ranked modifier genes, with *PMS1* exhibiting the most favourable profile. Notably, *HTT* itself ranked poorly as a theoretical drug target, emphasizing the importance of exploring modifier-based alternative targets. In conclusion, our study highlights the importance of human genomic information to prioritize Huntington’s disease modifier genes as drug targets, providing a basis for future therapeutic development in Huntington’s disease and other repeat expansion disorders.

## Teaser

Using diverse human genetic evidence, Namuli *et al*. investigated age-of-onset modifier genes in Huntington’s disease as potential drug targets. This highlighted that, for repeat expansion disorders, preclinical therapeutic research should focus on mismatch repair genes, such as *PMS1*, as alternate approaches to the traditional targeting of pathogenic genes.

## Graphical Abstract

**Graphical Abstract:**
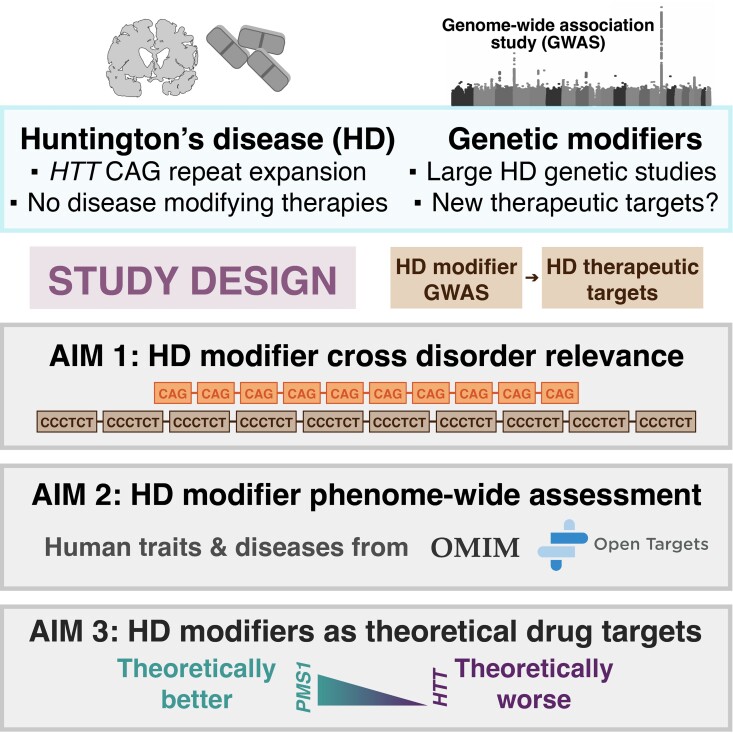

## Introduction

Huntington’s disease is a fatal autosomal dominant disorder caused by a CAG repeat expansion in the huntingtin gene, *HTT.*^1^ Although the exact mechanisms remain to be delineated, this mutation leads to processes that result in neuronal cell death, primarily in the cortical and striatal regions of the brain.^2^ Individuals with Huntington’s disease experience changes in cognitive function, psychiatric disturbances and motor skill impairment, typically with an average age of onset (AOO) of around 45 years.^2^ Disease onset is inversely correlated with the size of the CAG repeat expansion, but this relationship accounts for 70% of the variability observed among affected individuals.^3^ For example, individuals with the same CAG repeat size can first exhibit symptoms, sometimes decades apart.^4^ This suggests the existence of additional factors that contribute to differences in AOO and disease severity.

Genetic modifiers can explain some of these differences.^3^ Modifiers are genes that do not directly cause the disease but influence its phenotypic outcomes, including the AOO and disease severity.^5^ Modifier genes are of considerable interest as they can expand the therapeutic targets for rare diseases beyond the pathogenic mutations/genes. This is especially important in Huntington’s disease due to the recent halting of *HTT*-lowering clinical trials for both allele-specific and non-selective approaches.^6^ Further, the therapeutic impact of targeting Huntington’s disease modifiers could be broadened if underlying modifier effects are shared across >50 repeat-mediated disorders.^4^

Notably, recent genome-wide association studies (GWASs) have successfully identified genetic modifiers that influence the AOO in Huntington’s disease by analysing thousands of affected individuals.^7^ Among these modifiers are several genes related to DNA mismatch repair, including *FAN1*, *LIG1*, *MLH1*, *MSH3*, *PMS1* and *PMS2*,^7^ which are thought to impact the somatic expansion of the CAG repeat in vulnerable neural cell types. These DNA-repair-related GWAS signals were easily annotated and resolved due to the known, but previously underappreciated role of somatic repeat instability in Huntington’s disease pathogenesis. However, several loci arising from Huntington’s disease AOO GWAS remain unresolved, and their mechanistic basis is unknown, limiting their inclusion as therapeutic targets.

To bridge this knowledge gap, as well as to learn more about Huntington’s disease modifier genes, large-scale repositories of human genomic data can be used. By studying the results from thousands of unbiased genome-wide screens in humans, we can gain new insights into shared biological processes, which may be relevant to future functional studies, and identify potential adverse consequences of targeting these genes therapeutically. Further, recent research evaluating past clinical trial success is identifying new factors associated with successful drug development in addition to prior human genetic evidence.^8-11^ This information is valuable as most candidate therapeutics fail (i.e. >90%) after lengthy and costly clinical trials.^12^ We, therefore, set out to leverage diverse human genomic information to prioritize Huntington’s disease modifier genes for preclinical study and assess the cross-repeat-expansion disorder relevance of these related variants. Our analyses, which focus on robust human genetic evidence, provide important information for repeat expansion disorder therapeutic development.

## Materials and methods

### Assigning Huntington’s disease genetic modifier GWAS loci to genes and exploring shared relevance in other repeat expansion disorders

Ethical clearance was obtained from the local Research Ethics Board (H2022:354 HS25743). We analysed summary statistics from a large Huntington’s disease AOO GWAS.^7^ This contains information from 9064 individuals affected by Huntington’s disease (4417 males and 4674 females) and 21 genome-wide significant loci in addition to the original signals from *HTT* CAG interrupting sequence variants.

To annotate Huntington’s disease modifier loci, we used the Open Targets Genetics (OTG) Variants to Gene (V2G) function using the top GWAS variants. Briefly, this function aggregates genomic information from diverse sources to map V2G.^13^ This information includes data from quantitative trait loci, chromatin interactions, *in silico* variant predictions and distance from transcription start sites. Genes with the top V2G scores at each modifier locus were prioritized for analysis.

In subsequent analyses, we focused on the most robust loci, excluding those supported by only a single rare variant in the GWAS analyses.

We also assessed shared evidence for these genes in other Huntington’s disease/huntingtin modifier screens, including human sequencing and animal model studies. We evaluated a recent Huntington’s disease AOO exome sequencing study^14^ to identify Huntington’s disease modifier genes that showed at least nominal (*P* < 0.05) support for differentially harbouring predicted loss of function variants or highly deleterious variants [i.e. non-synonymous and predicted damaging to protein function; combined annotation–dependent depletion score ≥ 20 (NSD20) analysis, top 1% of most damaging] for Huntington’s disease phenotypes. Finally, we confirmed whether any GWAS-related modifiers were potentially involved in mHtt toxicity using data from unbiased genome-wide scans in mice.^15^

To inform cross-disorder modifier relevance, we empirically evaluated the effect sizes of the top GWAS signals related to Huntington’s disease AOO in summary statistic data from a GWAS (*n* = 353) of age-related penetrance of the repeat expansion disorder, X-linked dystonia-parkinsonism (XDP).^16^ These results included variants with minor allele frequency (MAF) > 1%, which includes variants not captured in the original study (previous MAF cut-off 5%). The effect sizes of overlapping XDP GWAS variants with top Huntington’s disease modifier signals were analysed using linear regression to determine correlation across traits using linear regression in R. These results were visualized using a *ggplot2* scatter plot with a fitted linear model [i.e. stat_smooth(method=‘lm’)], further annotating whether individual variants were significant (*P* < 0.05) in the XDP GWAS data.

### Profiling Huntington’s disease modifier signals for association with human diseases and traits

To gain insight into the potential off-target effects of therapeutically perturbing Huntington’s disease modifier genes, we systematically studied modifier-related associations with rare and common diseases, as well as human traits. For severe rare diseases, we programmatically queried two curated resources: ClinVar and the Online Mendelian Inheritance in Man (OMIM) databases.^17,18^ We extracted and analysed Huntington’s disease modifier information from: (i) the gene-specific ClinVar summary (accessed: May 2022), representing 2 694 381 total alleles (219 473 reported alleles reported as pathogenic or likely pathogenic) and the OMIM genemap2 (accessed: June 2022) consisting of 5779 phenotype entries.

Next, we performed a comprehensive phenome-wide study to discover common human diseases and traits that can also be attributed to Huntington’s disease modifier genes by analysing data from the OTG database (version 8).^13^ This repository contains information from 57 244 studies, including data from 8894 studies with full GWAS summary statistics. In brief, we accessed (February 2023) the OTG application programming interface using the *ghql* package and converted JSON data to R objects using the *jsonlite* package. We utilized the locus-to-gene (L2G) gene prioritization tool to determine Huntington’s disease modifier genes likely to drive the underlying GWAS signals.^13^ L2G uses machine learning techniques, trained on extensive genetic and functional data sets, to quantify the strength of evidence for trait–gene associations, with scores ranging from 0 to 1, with higher scores indicating increased confidence in causal gene identification. We considered L2G scores >0.5 to indicate evidence for the trait–disease association likely caused by the candidate Huntington’s disease modifier gene. We removed count-based terms (e.g. blood cell type measurements) from further analysis, as previously described.^19^ We used the LDLink SNPclip tool to identify unique haplotype associations (*R*^2^ > 0.5 in the European ancestry 1000 Genomes populations)^20^ when multi-variant trait associations were detected at a single modifier gene. Further, we used this approach to identify linkage disequilibrium between lead OTG trait variants and lead Huntington’s disease AOO variants.

### Assessing human genetic evidence and providing an evaluation of theoretical druggability for prioritized Huntington’s disease modifier genes

#### Human genetic evidence

To assess the human genetic evidence related to the prioritized candidate Huntington’s disease modifier genes and to identify those with the most robust support, which is essential for future preclinical studies, we collected data from various sources, including phenotypes relevant to Huntington’s disease and repeat expansion disorders. We annotated the number of independent hits at each gene locus to further incorporate Huntington’s disease AOO GWAS data. We also annotated whether the candidate modifier genes have cross-repeat expansion disorder support from our analyses of the XDP-modifier GWAS,^16^ as well as a polyglutamine disorder modifier study that used targeted genotyping.^21^ Since somatic instability of the *HTT* CAG repeat likely contributes to Huntington’s disease AOO, we documented whether lowering the related modifier genes has been associated with changes in instability in human pluripotent stem cells (hPSCs) or striatal neurons derived from these cells.^22,23^ We also documented severe disease information from our prior OMIM analyses to capture potential off-target effects.

#### Theoretical druggability

We evaluated the theoretical druggability of the prioritized Huntington’s disease modifier genes by examining diverse features. Based on the direction of effects for Huntington’s disease AOO GWAS signals and exome data, as well as the literature, we indicated whether knockdown would theoretically be beneficial in Huntington’s disease since this is more readily achieved therapeutically than overexpression. We also included evidence for druggability based on the Drug Gene Interaction Database ‘druggable any’ category (accessed: June 2023).^24^ We also assessed drug target profile favourability using recently identified features associated with past clinical trials ending due to safety concerns or adverse drug reactions.^10^ We used metrics identified in the original study to classify profiles as either favourable or unfavourable. Specifically, unfavourable metrics, identified and defined by Razuvayevskaya *et al.*, were: (i) intolerant to loss-of-function variation [i.e. probability of being loss-of-function intolerant (pLI) > 0.9 from the Genome Aggregation Database (gnomAD) v2.1.1 data set, accessed: July 2022], (ii) numerous interacting partners (i.e. >10 partners with mi score >0.42 from the IntAct database, accessed: July 2022) and (iii) broad tissue expression (i.e. low tissue specificity in the Human Protein Atlas, February 2023).

Finally, to systematically query and identify both approved drugs and those in clinical development and to further assess clinical and discovery precedence, we used the recently developed tool, OncoEnrichR,^25^ which collates information from the Open Targets Platform and the NCI Thesaurus.

### Statistical analyses

All statistical analyses were performed in R v4.3 and implemented in the RStudio Server using *tidyverse* principles and related packages. Linear regression was used to determine the overlap between the effect sizes of top Huntington’s disease AOO GWAS signals with XDP GWAS effect sizes. For disease and trait associations with modifier genes, we used L2G scores >0.5 to link GWAS trait signals to candidate modifier genes. Statistical analyses are described in further detail in the relevant methods sections above and can be found in the study-specific code repository (see Data availability).

## Results

### Bioinformatic prioritization of Huntington’s disease modifier genes and overlap of effects in another repeat expansion disorder

**Figure 1:**
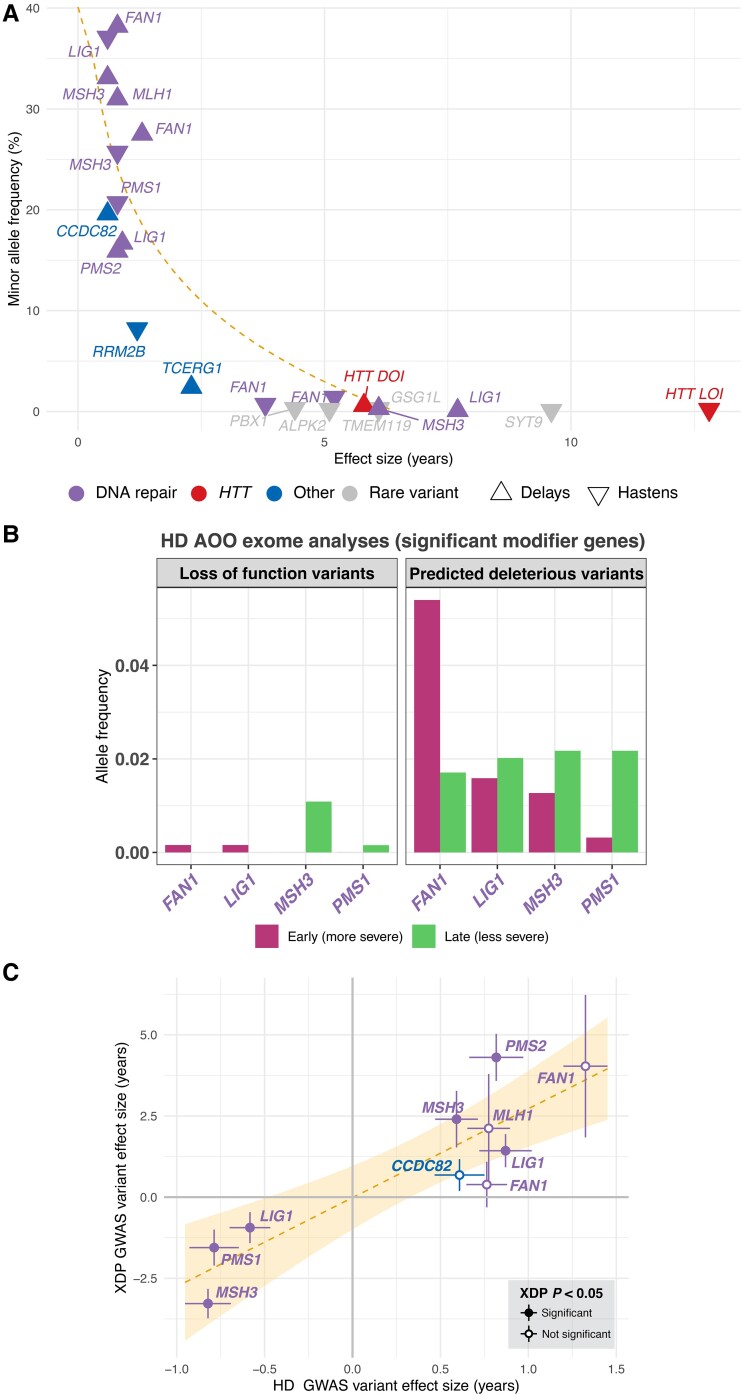
**Huntington’s disease AOO-associated variants display a broad spectrum of effect sizes and have potential cross-repeat expansion disorder relevance.** (**A**) The variant effect size (i.e. years earlier/later) of Huntington’s disease AOO GWAS hits is inversely correlated to allele frequency. A rare tag variant for the *HTT* loss of CAA interruption (LOI) displays the greatest impact on the age of onset, while common variants with allele frequencies of >25% have a more subtle impact on this trait (i.e. <2 years, on average) but are carried by more affected individuals. Prioritized candidate genes for each respective variant have been labelled and annotated by pathway and effect direction for that variant. The dashed line represents the linear regression of log-transformed AOO effect size on MAF of the 21 genome-wide significant variants assessed. (**B**) Combined allele frequency of loss of function and predicted deleterious variants in an exome sequencing data set of AOO in Huntington’s disease stratified by very early onset (*n* = 250) and very late onset (*n* = 250). Only Huntington’s disease modifier genes with evidence for association with onset time (*FAN1*, *LIG1*, *MSH3* and *PMS1*) are plotted. (**C**) Overlapping Huntington’s disease AOO GWAS hits show similar effect sizes in a GWAS of age-associated penetrance of XDP, a repeat expansion disorder caused by a different motif (i.e. CCCTCT) in the *TAF1* gene. These results are highly correlated (*R*^2^ = 0.78, linear regression *P* = 7.3 × 10^−4^), suggesting a shared effect between these two repeat expansion disorders. Bars extending from each point represent standard error. The dashed line represents the linear regression of XDP effect size on Huntington’s disease AOO effect size, with the yellow-shaded area representing the 95% confidence interval for the regression line. Points are filled based on whether there was a significant association (linear regression *P* < 0.05) with XDP AOO in the related GWAS (*n* = 353).

We identified and annotated 14 candidate Huntington’s disease modifier genes independent of *HTT* interrupting sequence signals that may play a role in modifying the AOO in Huntington’s disease (Fig. 1A, Supplementary Table 1). To assess the reliability of our gene prioritization strategy, we focused on the DNA-repair-related GWAS signals, where causal genes have likely been identified. Out of 13 variants related to DNA repair signals, 11 were correctly identified as belonging to these genes, resulting in a sensitivity (true positive rate) of 84.6%. For subsequent analyses, we reassigned incorrectly mapped *LRRFIP2* rs1799977 to *MLH1* and *CCZ1* rs74302792 to *PMS2*. After these corrections, resolved genes were assigned to DNA repair pathways (i.e. *FAN1*, *LIG1*, *MLH1*, *MSH3*, *PMS1* and *PMS2*) and other pathways (i.e. *CCDC82*, *RRM2B*, *TCERG1* and *TMEM119*), excluding single rare variant association signal genes (i.e. *ALPK2*, *GSG1L*, *PBX1* and *SYT9*).

The effect size of individual modifier GWAS variants was inversely correlated with MAF in the Huntington’s disease cohort (Fig. 1A). Re-analysis of Huntington’s disease AOO exome sequencing data showed that, in the early onset Huntington’s disease group, deleterious variants were enriched in *FAN1* and depleted in *LIG1*, *MSH3* and *PMS1* (Fig. 1B). Further, *CCDC82* and *TCERG1* also overlapped with hits from a genome-wide modifier screen of mHTT toxicity in zQ175 mice. Notably, when assessing the overlap between top Huntington’s disease modifier variants found within XDP repeat expansion disorder GWAS data (*n* = 10 variants), a high correlation between Huntington’s disease AOO and XDP age-associated penetrance was observed (*R*^2^ = 0.78, *P* = 7.3 × 10^−4^; Fig. 1C). Variants included in these analyses were mainly annotated to DNA repair genes (*FAN1*, *MSH3*, *MLH1*, *LIG1*, *PMS2* and *PMS1*), in addition to *CCDC82*. *CCDC82*, *LIG1*, *MSH3*, *PMS1* and *PMS2* all displayed nominally significant associations (*P* < 0.05) in the XDP AOO GWAS, and it should be noted that variants related to *FAN1* and *RRM2B* have also previously been associated with AOO in polyglutamine disorders.^21^

### Diverse human diseases and traits are associated with Huntington’s disease modifier genes

**Figure 2:**
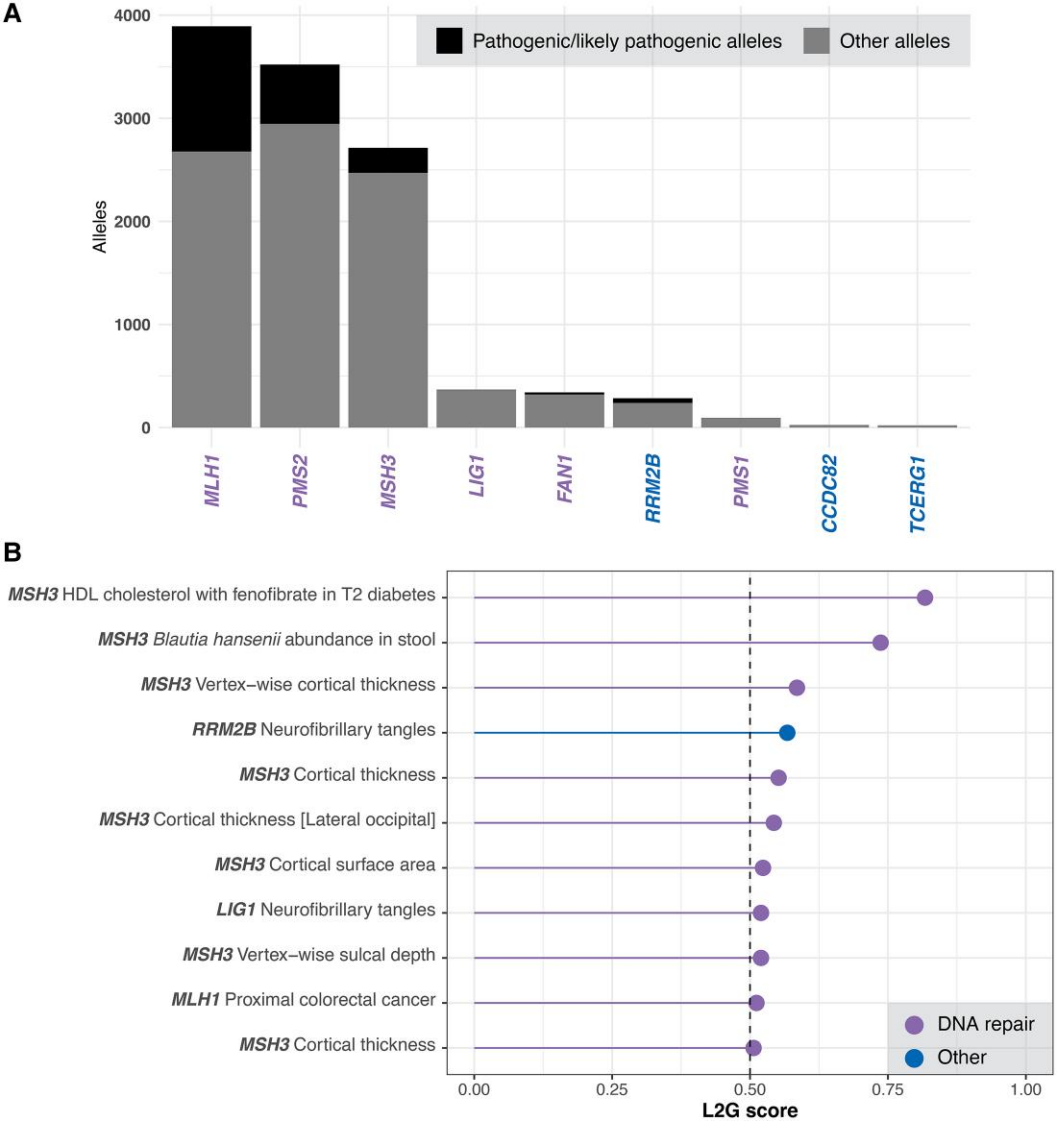
**Analysis of clinical genetic repositories and human GWASs can provide insights into potential unintended consequences of therapeutically targeting Huntington’s disease modifier genes and clarify their role in pathogenic processes.** (**A**) DNA repair genes, especially mismatch repair pathway members, *MLH1*, *PMS2* and *MSH3*, account for the majority of ClinVar pathogenic/likely pathogenic alleles due to their involvement in cancer. Therapeutically targeting these genes should be approached with caution since some mismatch repair deficiency syndromes are associated with the brain/central nervous system. (**B**) Prioritized gene–trait associations for Huntington’s disease modifiers that were identified through the analysis of the large repository of unbiased GWASs in the OTG database. Prioritized gene–trait pairs with Locus2Gene (L2G) >0.5 are plotted (i.e. indicating that the related GWAS trait signal is likely to arise from genetic variation linked to the Huntington’s disease modifier gene). The pairs identified include important neurobiological associations, including cortical thickness (*MSH3*) and neurofibrillary tangles (*LIG1* and *RRM2B*).

A total of five Huntington’s disease modifier genes (*FAN1*, *MLH1*, *MSH3*, *PMS2* and *RRM2B*) had OMIM phenotype entries, mainly for cancer-related diseases for DNA-repair-related genes (Table 1). We detected 11 254 alleles in the ClinVar database, 2108 (18.7%) of which were annotated as pathogenic/likely pathogenic. The DNA repair pathway also contributed most pathogenic/likely pathogenic alleles, particularly *MLH1*, *PMS2* and *MSH3*, which contributed 96.5% (*n* = 2034) of alleles of this kind. For our common human trait/disease-related analyses, we extracted 983 signals from GWAS for the 10 Huntington’s disease modifier genes from the OTG database (see Data availability for access to these associations), filtering these down to 39 signals from 6 genes using machine-learning-based gene prioritization (i.e. L2G scores >0.5). When removing count-based terms, we visualized 11 signals from four Huntington’s disease modifier genes (*MSH3 n* = 8, *LIG1 n* = 1, *MLH1 n* = 1, *RRM2B n* = 1; Fig. 2B), some of which are related to neurological processes that could be relevant to Huntington’s disease.

**Table 1:** Rare Mendelian diseases that are caused by rare mutations in Huntington’s disease modifier genes in the OMIM database

Gene	OMIM condition	Inheritance	Pathway
*FAN1*	Interstitial nephritis, karyomegalic	Autosomal recessive	DNA repair
*HTT*	Lopes–Maciel–Rodan syndrome	Autosomal recessive	*HTT*
*HTT*	Huntington’s disease	Autosomal dominant	*HTT*
*MLH1*	Colorectal cancer, hereditary nonpolyposis, Type 2	Autosomal dominant	DNA repair
*MLH1*	Mismatch repair cancer syndrome 1	Autosomal recessive	DNA repair
*MSH3*	Familial adenomatous polyposis 4	Autosomal recessive	DNA repair
*MSH3*	Endometrial carcinoma	Somatic	DNA repair
*PMS2*	Colorectal cancer, hereditary nonpolyposis, Type 4		DNA repair
*PMS2*	Mismatch repair cancer syndrome 4	Autosomal recessive	DNA repair
*RRM2B*	Mitochondrial DNA depletion syndrome 8A and B	Autosomal recessive	Other
*RRM2B*	Progressive external ophthalmoplegia with mitochondrial DNA deletions	Autosomal dominant	Other

Documented OMIM phenotypes are represented for each of these genes.

The eight gene–trait associations detected for *MSH3* arose from three independent signals. The main haplotype was linked to four variants (rs12517451, rs863216, rs245100 and rs1650697, *R*^2^ > 0.95) and was associated with several cortical morphology-related traits assessed using neuroimaging.^26-29^ This cortical morphology-related haplotype was also in strong linkage disequilibrium (i.e. *R*^2^ > 0.95) with one of the *MSH3* Huntington’s disease modifier signals (i.e. rs701383). The remaining *MSH3* signals were for pharmacogenomic (lead variant: rs7709909)^30^ and microbiome (lead variant: rs6151874)^31^ related traits, which have less clear relevance to Huntington’s disease. Both *LIG1* (lead variant: rs274876) and *RRM2B* (lead variant: rs2061299) were associated with the same neurobiological trait, neurofibrillary tangles.^32^ The *LIG1* rs274876 neurofibrillary tangle-associated variant is in high LD with the intronic Huntington’s disease AOO *LIG1* rs3730945 variant (*R*^2^ = 0.82), while the *RRM2B* signal was independent of the Huntington’s disease AOO *RRM2B* locus. Further, another *RRM2B* neurological disease progression trait association (i.e. rate of cognitive decline in mild cognitive impairment) approached our L2G threshold (L2G = 0.41, lead variant: rs7840202).^33^

### Human genetic evidence and theoretical druggability analyses of Huntington’s disease modifier genes prioritize therapeutic targets

**Figure 3:**
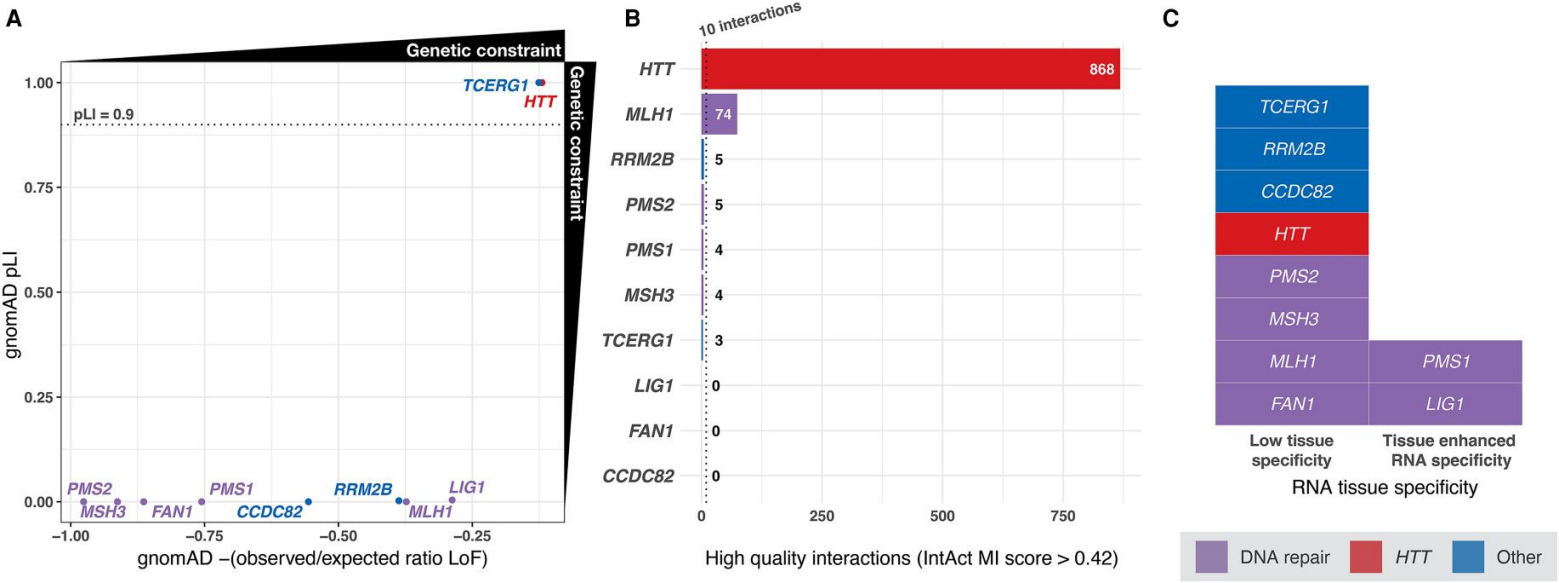
***HTT* and Huntington’s disease modifier genes display different metrics for genomic features associated with past clinical trial success. Relevant thresholds for classification as favourable targets are indicated with dashed lines.** (**A**) Genetic constraint metrics obtained from gnomAD [probability of being pLI and the negative observed/expected ratio for loss of function (LoF) variants] illustrate that both *HTT* and *TCERG1* are highly constrained. The pLI is known to be a dichotomous-like metric (https://gnomad.broadinstitute.org). Most genes can be classified as non-constrained (pLI < 0.1) or constrained (pLI > 0.9), as can be seen in the figure. We include another measure of constraint, which is more quantitative (−observed/expected ratio, negative transformation used to make comparisons with pLI on the plot more intuitive) to illustrate the most constrained genes further. (**B**) *HTT* displays significantly more interacting partners (*n* = 868) than Huntington’s disease modifier genes based on IntAct Molecular Interaction (MI) scores >0.42. (**C**) The majority of Huntington’s disease modifier genes, as well as *HTT*, display low tissue specificity for RNA expression, except for *PMS1* and *LIG1*, which demonstrate tissue-enhanced RNA specificity.

**Figure 4:**
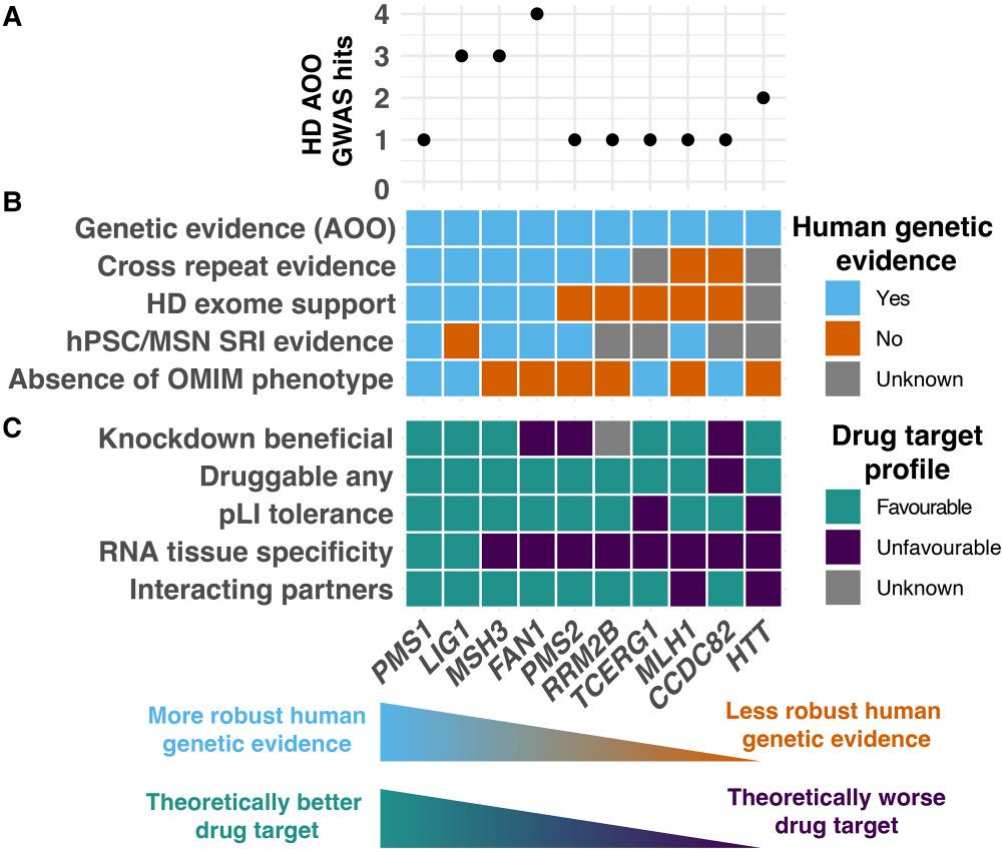
**Huntington’s disease genetic modifier drug target profiles can be used to assess theoretical druggability by providing diverse evidence related to clinical trial success.** Huntington’s disease modifier genes have been ordered based on the number of favourable scores obtained in our analyses. (**A**) Annotation of Huntington’s disease AOO GWAS hits by candidate modifier gene by number of independent GWAS hits and whether they are supported by only a rare variant association. Genes with multiple hits at a locus have more robust evidence for involvement with Huntington’s disease AOO. (**B**) Aggregated human genetic evidence relating to Huntington’s disease modifiers and *HTT* provides insights into the robustness of findings relating to these genes. Colours represent evidence in support (blue) or refutation (orange) for their involvement in relevant phenotypes/features, with lack of information displayed in grey. While *LIG1* has a large amount of human genetic support, crucially, it has not been shown to influence somatic repeat instability in human stem cell models of Huntington’s disease. (**C**) Ranking Huntington’s disease modifier genes by favourable (teal green) and unfavourable (deep purple) metrics based on criteria associated with clinical trial stoppage due to safety concerns provides an unbiased way to prioritize candidate Huntington’s disease modifier genes. Genes that are theoretically better targets to prioritize for future study are on the left. *PMS1* was ranked the most favourably for these factors. Notably, *HTT* ranked unfavourably for several criteria, which is in line with the recent failure of *HTT*-lowering trials. **A**, **B** and **C** share the same *x*-axis, representing the Huntington’s disease modifier gene name. hPSC, human pluripotent stem cell; MSN, medium spiny neuron; SRI, somatic repeat instability.

Using information from recent human genetics research and curated repositories, combined with predictive features for potential clinical trial success, allowed us to identify patterns that may inform future preclinical research (Figs. 3 and 4). *PMS1*, *LIG1*, *MSH3* and *FAN1* displayed the most robust human genetic evidence; however, modulating *LIG1* has not been shown to alter somatic repeat instability in hPSC models. Further, rare mutations in *MSH3* and *FAN1* are known to cause rare forms of cancer, although they do not primarily affect the central nervous system. We also summarized human genetic data to support whether modifier gene knockdown would be theoretically beneficial in Huntington’s disease (Supplementary Table 2). *FAN1*, *PMS2* and *CCDC82* would potentially require overexpression-based therapeutic approaches, while more research is required for *RRM2B* to determine the effect direction.

We developed comprehensive drug profiles by assessment of characteristics associated with past clinical trial stoppage due to safety concerns, allowing us to rank Huntington’s disease modifier genes based on these features (Fig. 4C). Notably, DNA repair pathway member, *PMS1*, ranked favourably for all criteria, followed by other mismatch repair genes *LIG1* and *MSH3*. Only *PMS1* and *LIG1* displayed favourable tissue specificity (tissue-enhanced expression). Since *HTT*-lowering therapeutics is the most mature area in Huntington’s disease therapeutics, we included *HTT* in this analysis for reference. Notably, *HTT* displayed unfavourable metrics, displaying characteristics associated with clinical trial stoppage due to safety concerns (i.e. pLI tolerance, RNA specificity and interacting partners). While we used thresholds identified in the original study to inform these decisions, plotting the numeric values for these metrics (Fig. 3) confirmed that *HTT* substantially surpasses these metrics, with 866 high-quality interactions (threshold: 10 interactions) and the most severe pLI constraint score possible of 1.0 (threshold: 0.9).

Finally, no approved drugs were identified for the Huntington’s disease modifier genes, and only *RRM2B* had therapeutics in clinical development. Specifically, *RRM2B* targets in late clinical development/phase (Phase 3–4; hydroxyurea, motexafin and gadolinium) and early clinical development/phase (Phase 1–2; gallium nitrate and triapine). Discovery precedence was available for *LIG1*, *MLH1*, *MSH3*, *PMS2* and *TCERG1*, and no antibody tractability (medium to high confidence) was identified for any of the prioritized Huntington’s disease modifier genes (Supplementary Table 3).

## Discussion

The lack of disease-modifying therapies for repeat expansion disorders has led to an increased therapeutic focus on large-scale Huntington’s disease genetic modifier GWAS. These transformative studies contain a wealth of information but require additional characterization to identify the modifier mechanisms underlying these signals and to assess the potential of related genes as therapeutic targets. We used a comprehensive approach focused on human genomic information to address these gaps and inform Huntington’s disease therapeutics. We confirmed the cross-repeat expansion disorder relevance of Huntington’s disease modifier variants and identified shared associations between modifier genes and other human traits and diseases. Our gene prioritization also improved confidence in potentially causal modifier genes. Finally, ranking of Huntington’s disease modifier genes based on diverse human genetic information and features that have been linked to past clinical trial success provides an objective way to prioritize genes as potential drug targets, therefore guiding the development of rational therapies.

Firstly, we performed a cross-repeat expansion disorder analysis since a shared modifier effect has potential broad-reaching consequences. Targeting shared processes, such as somatic repeat instability, could theoretically allow several disorders to be targeted with one approach. Shared repeat expansion disorder modifier mechanisms were initially suggested through an analysis of Huntington’s disease and polyglutamine spinocerebellar ataxias, where variants in *FAN1*, *PMS2* and *RRM2B* displayed significant associations.^21^ In our work, we assessed recent data from an XDP-modifier GWAS, a study highlighting the importance of *MSH3* and *PMS2*.^16^ Our results extend the scope of this finding and highlight the importance of other DNA repair genes underlying this signal, including *PMS1* and *LIG1*. Adding support for a broad repeat expansion disorder genetic modifier model is that shared effects are observed for variants from numerous modifier genes despite a different repeat motif for XDP (i.e. CCCTCT). Independent assessment of the Huntington’s disease AOO GWAS data revealed an inverse relationship between allele frequency and modifier effect size, highlighting the importance of performing larger GWAS to detect additional therapeutic targets. A current limitation of the XDP AOO GWAS and those of other repeat expansion disorders outside of Huntington’s disease is the limited sample sizes of these cohorts. This should be addressed in the future to expand discovery and predictive power. Further, similarities in repeat expansion disorder effects open the opportunity to perform cross-repeat expansion disorder meta-GWAS, increasing discovery power for identifying shared drug target genes.

Additional Huntington’s disease modifier support for DNA repair genes can also be found when assessing rare variants from human exome sequencing-based AOO studies, specifically for *FAN1*, *LIG1*, *MSH3* and *PMS1*. To expand on this further, we used a broad approach to identify and characterize Huntington’s disease modifier genes, including those outside mismatch/DNA repair pathways. Many of these unresolved genes are involved in neurological traits and some overlap with murine mHTT modifier screen results, substantiating their evidence for involvement in Huntington’s disease. Current Huntington’s disease modifier hypotheses suggest a two-hit process, where DNA-repair-related modifiers interact with somatic CAG repeat instability until a key CAG threshold is reached, triggering toxicity.^34^ Several unresolved modifier loci/genes are expected to fall within the toxicity category. For example, our reassessment of modifiers of mHTT toxicity in zQ175 mice showed an overlap with Huntington’s disease modifier genes *CCDC82* and *TCERG1*. The need for additional study of toxicity-related drivers is emphasized by the fact that the function of the *CCDC82* is currently unknown. However, *CCDC82* has been linked as a potential cause of rare cases of intellectual disability.^35^ Therapeutic strategies are limited as the exact toxicity processes remain undefined.

Analysis of clinical genetic repositories provides insights into potential unintended consequences of therapeutically targeting these modifier genes. Mismatch repair genes *MLH1*, *PMS2* and *MSH3* displayed an increased number of pathogenic alleles and were linked to cancer phenotypes, suggesting potential limitations as drug targets. However, the potential off-target consequences require careful evaluation of the risk–benefit profile since these cancers primarily affect the brain.

Studying the results of unbiased human genomic studies and identifying gene–trait pairs can help us identify important pathways and processes that Huntington’s disease modifiers participate in in relation to Huntington’s disease pathogenesis and mHTT toxicity. This can help inform future functional genomic studies to determine the underlying mechanisms. In this regard, our exploratory machine-learning-based analysis of over 133 000 GWAS loci identified multiple phenotypes associated with the modifier genes, including those related to neurodegenerative diseases (i.e. neurofibrillary tangles) and brain structure and morphology (e.g. cortical thickness/surface area). Notably, *LIG1* and *RRM2B* were independently associated with a GWAS of neurofibrillary tangles,^32^ suggesting a potential role in mHTT aggregates. Studying this phenotype with these genes should be prioritized for further study in Huntington’s disease using functional genomics. *RRM2B* could also be involved in the disease progression of other neurodegenerative disorders, such as Alzheimer’s disease, indicating potential cross-disorder relevance. We detected several cortical thickness/surface area traits pleiotropically linked to *MSH3*, arising from a shared haplotype with one of the modifier signals. Cortical thinning is observed in Huntington’s disease, even during the prodromal phases before disease onset,^36^ so understanding how *MSH3* contributes to cortical morphology in general and brain atrophy is important.

Our human genetic evidence aggregation and therapeutic analysis, which incorporated new predictors of clinical trial success, provides important information to prioritize Huntington’s disease modifier genes. *MSH3* has been the focus of the most intensive Huntington’s disease modifier therapeutic research but was not the top candidate since it displays broad RNA tissue expression. Mismatch repair gene *PMS1* displayed the most favourable profile, providing support for its additional therapeutic investigation and more comprehensive inclusion in future studies. This could include developing a *PMS1*-specific splice modulator for pseudo-exon inclusion.^37^ Knockout of either *Msh3* or *Pms1* in mHtt CAG 140 knock-in mice has recently been shown to alleviate molecular phenotypes in Huntington’s disease–vulnerable striatal neurons (e.g. lack of mHTT aggregates at 6 months),^38^ further illustrating potential therapeutic relevance. *LIG1* also ranked highly in our analyses, but importantly, lowering of *LIG1* in 125Q Huntington’s disease hPSCs^22^ and Huntington’s disease knock-in mice^39^ has not been shown to impact somatic instability of the CAG repeat. This may arise due to ligase functional redundancy in humans and mice,^22,39^ indicating the importance of assessing genetic evidence from mouse models and human genetic studies. Further dampening therapeutic support for *LIG1* is that there is evidence that biallelic mutations in the gene are linked to immune deficiencies.^40^*FAN1* also scored relatively favourably in our analyses, but any repeat expansion disorder-related therapy with this gene would require an upregulation-based approach. This may be challenging, although newer RNA-targeted therapeutics^41^ may prove helpful in this area. Additionally, mismatch repair members outside of current GWAS hits, such as *MLH3*, could also be assessed in the future.^42^ Adverse safety profiles should still be evaluated, especially for DNA repair genes, mainly since oncology-related targets are also associated with risks.^10^ Finally, the identified *RRM2B*-related ribonucleotide reductase inhibitors in clinical development as cancer treatments may also have safety concerns as neurological disease treatments.

A key result from our analyses is that *HTT* ranked poorly as a theoretical drug target, thus highlighting the importance of exploring alternate therapeutic targets, especially considering recent failures of *HTT*-lowering trials. For our analyses, we used thresholds determined by a prior study of factors associated with past clinical trial stoppage due to safety concerns (i.e. constraint, gene expression fidelity and interacting partners).^10^ While these thresholds are binary, *HTT* has 85 times more interacting partners than this threshold and displays maximum pLI constraint, and broad tissue/cell type expression of the gene is well documented. When considering lowering strategies, maintaining certain physiological levels of *HTT* is important—hypomorphic *HTT* mutations cause a neurodevelopmental disorder, LOMAR syndrome,^43^ and common *HTT* genetic variation linked to ageing and cognitive traits.^19^ The results of a new paradigm-changing study that performed paired single-cell gene expression and CAG-repeat length analysis in human Huntington’s disease brains also bring the field’s focus on *HTT* lowering under scrutiny.^44^ Firstly, striatal neuron loss did not correlate with *HTT* expression levels. This study also showed that vulnerable striatal neurons in Huntington’s disease accumulate somatic expansions over time, but there is no evidence of a harmful impact of expanded CAG repeat/mutant *HTT* until it reaches a somatic threshold of ∼150 CAGs late in life. Striatal neurons in affected individuals were estimated to spend over 95% of their lifetime below this threshold, and the harmful influence of mHTT only occurs during a short period before neuronal death. This potentially leaves a small window for *HTT* lowering to be effective compared with somatic repeat instability/mismatch repair-based approaches, which may have a larger therapeutic window.

## Conclusion

Our study focuses on the most robust Huntington’s disease modifier genes and provides important prioritization data for preclinical Huntington’s disease therapeutic studies based on diverse human genetic evidence. These results support the prioritization of specific DNA maintenance and repair genes, particularly mismatch repair genes *PMS1* and *MSH3*, for additional research. Therapeutically targeting Huntington’s disease modifiers provides the opportunity to develop disease-modifying therapies backed by causal human genetic evidence rather than using approaches that may only treat the symptoms. Although the effect sizes for variants related to these modifier genes identified by GWAS may be small, prior studies have shown that GWAS effect sizes do not necessarily correspond to therapeutic effects, which can be more substantial.^8^ As a next step, future functional genomics studies could include new human disease models for validation, such as 3D organoids,^45^ which could also be used to study the mechanisms of toxicity-driving modifiers. Further, it is important to note that poor performance in the current analysis does not equate to abandonment in future investigations and that all modifiers assessed here display human genetic support, which is associated with clinical trial success. For example, recent gene therapy–based *HTT*-lowering approaches in Phase I/II clinical trials have shown encouraging results.^46^ Ultimately, more than one modality/therapeutic target may be required to treat individuals affected by Huntington’s disease using combination therapy efficiently, and the pipeline implemented here could be combined with advanced fine-mapping techniques and used to study newly identified modifiers in larger GWAS cohorts as they become available.^47^

## Supplementary Material

fcae418_Supplementary_Data

## Data Availability

The data sets used in this study are publicly available or can be accessed by researchers through the external consortia that generated these data. Other relevant data and codes used in the current investigation are available at: https://github.com/Wright-Lab-Neurogenomics-Research/hd_mod_drug_target_analyses.
