# Supplementary material for: Genomic characterization of Huntington’s disease genetic modifiers informs drug target tractability
Source: Brain Commun. 2025 Jan 11;7(1):fcae418. doi: 10.1093/braincomms/fcae418 (PMC11724427; doi:10.1093/braincomms/fcae418)
Supplement: fcae418_Supplementary_Data [file fcae418_supplementary_data.pdf]

SUPPLEMENTARY INFORMATION

Supplementary Table 1. Annotation of HD age-of-onset GWAS loci for candidate modifier genes and related putative impact on HD. Locus names, variant effect sizes, and P-values collected from original study (GeM-HD 2019 *Cell*).

Chr	Position[a]	Locus[b]	Variant	Annotation[c]	Predicted	Resolved	MAF	*P*-value	Years[d]	Single rare variant
1	164283625	1AM1	rs567500111	Intergenic	*PBX1*	*PBX1*	0.3	6.90E-06	-4.4	Yes
2	190639915	2AM1	rs3791767	3’UTR	*PMS1*	*PMS1*	20.7	6.30E-08	-0.8	No
3	37053568	3AM1	rs1799977	Missense	*LRRFIP2*	*MLH1*	31	5.10E-10	0.8	No
4	2697660	4AM1	rs764154313	Intron	NA	*HTT*	0.2	2.10E-19	-12.8	No
4	2971698	4AM2	rs183415333	Intron	NA	*HTT*	0.6	1.40E-14	5.8	No
5	79913275	5AM1	rs701383	Intron	*MSH3*	*MSH3*	25.7	5.50E-10	-0.8	No
5	80086504	5AM2	rs113361582	Intron	*MSH3*	*MSH3*	0.3	1.30E-09	6.1	No
5	79990883	5AM3	rs1650742	Intron	*MSH3*	*MSH3*	33.1	1.60E-06	0.6	No
5	145886836	5BM1	rs79727797	NCT exon	*TCERG1*	*TCERG1*	2.4	3.80E-10	2.3	No
7	6079993	7AM1	rs74302792	Intron	*CCZ1*	*PMS2*	15.9	7.40E-08	0.8	No
8	103213640	8AM1	rs79136984	Downstream	*RRM2B*	*RRM2B*	8.2	3.60E-09	-1.2	No
11	96106737	11AM1	rs7936234	Intron	*CCDC82*	*CCDC82*	19.6	1.70E-05	0.6	No
11	7303052	11BM1	rs79714630	Intron	*SYT9*	*SYT9*	0.1	1.10E-08	-9.6	Yes
12	108992727	12AM1	rs140253376	Upstream	*TMEM119*	*TMEM119*	0.2	8.30E-06	-6.1	Yes
15	31202961	15AM1	rs150393409	Missense	*FAN1*	*FAN1*	1.4	1.80E-28	-5.2	No
15	31241346	15AM2	rs35811129	Intron	*FAN1*	*FAN1*	27.5	9.40E-26	1.3	No
15	31197995	15AM3	rs151322829	Missense	*FAN1*	*FAN1*	0.7	1.40E-08	-3.8	No
15	31230611	15AM4	rs34017474	Intron	*FAN1*	*FAN1*	38.2	8.50E-11	0.8	No
16	27873637	16AM1	rs187055476	Intron	*GSG1L*	*GSG1L*	0.3	5.50E-09	-6.1	Yes
18	56126806	18AM1	rs530017366	Intergenic	*ALPK2*	*ALPK2*	0.2	1.20E-04	-5.1	Yes
19	48622545	19AM1	rs274883	Intron	*LIG1*	*LIG1*	16.7	5.30E-09	0.9	No
19	48645976	19AM2	rs3730945	Intron	*LIG1*	*LIG1*	37.1	5.80E-07	-0.6	No
19	48620943	19AM3	rs145821638	Missense	*LIG1*	*LIG1*	0.1	1.50E-06	7.7	No

[a]hg19 position; [b]GeM-HD locus annotation; [c]Ensembl VEP annotation; [d]Effect of variant; [e]Predicted effect of increased expression/activity of the related modifier gene on HD onset. Abbreviations: Chr, chromosome; MAF, minor allele frequency; NA, not applicable; NCT, noncoding transcript; PMID, PubMed ID; UTR, untranslated region.

Supplementary Table 2. Summary of key evidence relating to the impact of theoretical knockdown of modifier genes on HD AOO and related traits. Human genetic evidence was prioritized within this summary.

Gene	Knockdown beneficial	Evidence
CCDC82	No	The top genome-wide significant GeM-HD GWAS variant is also an eQTL for *CCDC82* gene expression in the cortex, caudate, and putamen.[1] The HD AOO effect allele is associated with later onset and increased *CCDC82* expression.
FAN1	No	TWAS analyses of GeM-HD GWAS data have shown that increased cortical expression of *FAN1* is associated with delayed onset.[2] Exome analyses of HD AOO have shown that loss-of-function *FAN1* variants are associated with earlier onset in HD.[3] Loss of *FAN1* is associated with increased *HTT* CAG repeat instability in HD stem cell-derived striatal models.[4]
LIG1	Yes	A genome-wide significant GeM-HD *LIG1* GWAS variant is a predicted deleterious missense change (CADD 23.8) and results in later onset.[1] Exome analyses of HD AOO have shown that deleterious *LIG1* variants are nominally enriched in a the later AOO HD group.[3]
MLH1	Yes	Reduction of *MLH1* expression is associated with reduced somatic repeat instability in human HD stem cells striatal neuron cultures.[5]
MSH3	Yes	Exome analyses of residual HD AOO have shown that loss-of-function *MSH3* variants were exclusively found in late onset in HD group.[3] Reduction of *MSH3* expression is associated with reduced somatic repeat instability in human HD stem cells.[5]11/7/2024 10:38:00 PM
PMS1	Yes	Exome analyses of HD AOO have shown that deleterious *MS11* variants are enriched in a the later AOO HD group.[3] Reduction of *PMS1* expression is associated with reduced somatic repeat instability in human HD stem cells striatal neuron cultures.[5]
PMS2	No	TWAS analyses of GeM-HD GWAS data have shown that increased expression of *PMS2* in the brain is associated with delayed onset.[2] Contradictory evidence for the role of *PMS2* in somatic repeat expansion, with the gene being shown to both promote and reduce somatic CAG repeat expansion.[5,6]
RRM2B	Unknown	Different evidence relating to influence of gene expression on HD AOO effect for different TWAS tissues.[2] Warrants further research.
TCERG1	Yes	Focused TWAS sub-analysis of *TCERG1* gene expression indicates that increased cortical gene expression may be associated with earlier HD onset.[7]

AOO, age of onset; CADD, Combined Annotation Dependent Depletion; eQTL, expression quantitative trait locus; HD, Huntington disease; TWAS, transcriptome wide association study.

Supplementary references

1. GeM-HD Consortium. CAG Repeat Not Polyglutamine Length Determines Timing of Huntington's Disease Onset. *Cell*. 2019;178(4):887-900.e14. doi:10.1016/j.cell.2019.06.036

2. Wright GEB, Caron NS, Ng B, et al. Gene expression profiles complement the analysis of genomic modifiers of the clinical onset of Huntington disease. *Hum Mol Genet*. 2020;29(16):2788-2802. doi:10.1093/hmg/ddaa184

3. McAllister B, Donaldson J, Binda CS, et al. Exome sequencing of individuals with Huntington's disease implicates FAN1 nuclease activity in slowing CAG expansion and disease onset. *Nat Neurosci*. 2022;25(4):446-457. doi:10.1038/s41593-022-01033-5

4. Goold R, Flower M, Moss DH, et al. FAN1 modifies Huntington's disease progression by stabilizing the expanded HTT CAG repeat. *Hum Mol Genet*. 2019;28(4):650-661. doi:10.1093/hmg/ddy375

5. Ferguson R, Goold R, Coupland L, Flower M, Tabrizi SJ. Therapeutic validation of MMR-associated genetic modifiers in a human ex vivo model of Huntington disease. *Am J Hum Genet*. 2024;111(6):1165-1183. doi:10.1016/j.ajhg.2024.04.015

6. Pinto RM, Murtha R, Azevedo A, et al. Identification of genetic modifiers of Huntington's disease somatic CAG repeat instability by in vivo CRISPR-Cas9 genome editing. Published online June 9, 2024:2024.06.08.597823. doi:10.1101/2024.06.08.597823

7. Lobanov SV, McAllister B, McDade-Kumar M, et al. Huntington's disease age at motor onset is modified by the tandem hexamer repeat in TCERG1. *Npj Genomic Med*. 2022;7(1):1-9. doi:10.1038/s41525-022-00317-w

Supplementary Table 3. Tractability assessment of candidate HD modifier genes for small molecule and antibody targeting. OncoEnrichR was used to access relevant information from the Open Targets Platform and the NCI Thesaurus.

**Gene**	**Small molecule tractability category**	**Small molecule tractability support**	**Antibody tractability category**	**Antibody tractability support**
CCDC82	Unknown	Not applicable	Unknown	Not applicable
FAN1	Unknown	Not applicable	Unknown	Not applicable
LIG1	Discovery precedence	Structure with ligand | High-quality ligand | Druggable family	Unknown	Not applicable
MLH1	Discovery precedence	Structure with ligand | Medium-quality pocket	Unknown	Not applicable
MSH3	Discovery precedence	Structure with ligand	Unknown	Not applicable
PMS1	Unknown	Not applicable	Unknown	Not applicable
PMS2	Discovery precedence	Structure with ligand	Unknown	Not applicable
RRM2B	Clinical precedence	Advanced clinical | Med-quality pocket | Druggable family	Unknown	Not applicable
TCERG1	Discovery precedence	Structure with ligand	Unknown	Not applicable
